# Predicting Personality and Psychological Distress Using Natural Language Processing: A Study Protocol

**DOI:** 10.3389/fpsyg.2022.865541

**Published:** 2022-04-07

**Authors:** Jihee Jang, Seowon Yoon, Gaeun Son, Minjung Kang, Joon Yeon Choeh, Kee-Hong Choi

**Affiliations:** ^1^School of Psychology, Korea University, Seoul, South Korea; ^2^Department of Software, Sejong University, Seoul, South Korea; ^3^KU Mind Health Institute, Korea University, Seoul, South Korea

**Keywords:** personality prediction, psychological distress prediction, natural language processing, machine learning, the five-factor model of personality

## Abstract

**Background:**

Self-report multiple choice questionnaires have been widely utilized to quantitatively measure one’s personality and psychological constructs. Despite several strengths (e.g., brevity and utility), self-report multiple choice questionnaires have considerable limitations in nature. With the rise of machine learning (ML) and Natural language processing (NLP), researchers in the field of psychology are widely adopting NLP to assess psychological construct to predict human behaviors. However, there is a lack of connections between the work being performed in computer science and that of psychology due to small data sets and unvalidated modeling practices.

**Aims:**

The current article introduces the study method and procedure of phase II which includes the interview questions for the five-factor model (FFM) of personality developed in phase I. This study aims to develop the interview (semi-structured) and open-ended questions for the FFM-based personality assessments, specifically designed with experts in the field of clinical and personality psychology (phase 1), and to collect the personality-related text data using the interview questions and self-report measures on personality and psychological distress (phase 2). The purpose of the study includes examining the relationship between natural language data obtained from the interview questions, measuring the FFM personality constructs, and psychological distress to demonstrate the validity of the natural language-based personality prediction.

**Methods:**

Phase I (pilot) study was conducted to fifty-nine native Korean adults to acquire the personality-related text data from the interview (semi-structured) and open-ended questions based on the FFM of personality. The interview questions were revised and finalized with the feedback from the external expert committee, consisting of personality and clinical psychologists. Based on the established interview questions, a total of 300 Korean adults will be recruited using a convenience sampling method *via* online survey. The text data collected from interviews will be analyzed using the natural language processing. The results of the online survey including demographic data, depression, anxiety, and personality inventories will be analyzed together in the model to predict individuals’ FFM of personality and the level of psychological distress (phase 2).

## Introduction

### Background

Technological advances brought numerous changes in analyzing and predicting data in the field of psychology. In particular, the recent fourth industrial revolution and the development of computer technology made it possible to quickly and accurately analyze and predict human characteristics, with further innovations taking place. While many other fields (medicine, marketing, engineering, etc.) are rapidly integrating computer skills to develop technology that can be utilized in real life, the application of technology in the field of psychology remains limited and steady (Ahmad et al., 2020; Le Glaz et al., 2021). Many attempts are being made to evaluate and identify the psychological state or characteristics of an individual. However, research and technological development face challenges due to numerous theories and immense structures of personality (Zunic et al., 2020).

#### Traditional Personality Assessment

Understanding individuals’ personality gives substantial information about how people behave and adapt to the world. Personality psychology theories made attempts to explain human personality in a concrete and valid way, through accurately measuring individuals’ personality. In the field of clinical psychology and psychiatry, classifying personality disorders using personality measurements is a central objective. Disorders in personality have been categorically understood within the diagnostic system for a long time and assessing the presence or absence of the disorder has been an important topic. However, this method poses problems such as heterogeneity within the same category because the boundaries between disabilities are unclear, or having one disorder belonging to two or more categories at the same time (Widiger and Trull, 2007; Livesley, 2010). In addition, the inter-rater reliability among experienced clinical psychologists and/or psychiatrists diagnosing personality disorders using the categorical approach did not reach a sufficient level (Kraemer et al., 2012; Esbec and Echeburúa, 2015). For this reason, a dimensional model, which understands personality as a complex hierarchy of continuously distributed attributes rather than a categorical approach, is receiving attention in understanding personality and diagnosing disorders (Costa and McCrae, 2017). Many empirical studies have proven its validity and usefulness (Widiger, 2017). The Five-Factor Model (FFM), which explains personality with Neuroticism, Extraversion, Openness, Agreeableness, Conscientiousness, and their many facets, is a well-known dimensional model of personality (McCrae and John, 1992).

Traditionally, a self-report multiple choice questionnaires have been widely utilized to quantitatively measure one’s personality and other psychological constructs. This measure has extreme practicality in that it simply requires the target person’s participation and can readily collect sufficient information in one sitting (Paulhus and Vazire, 2007). Despite other definite strengths (e.g., brevity and utility), the self-report multiple choice questionnaires have several limitations in nature. First, it is possible for respondents to hide or distort their responses, especially in the context of forensic or evaluation settings for employment (White et al., 2008; Fan et al., 2012). To prevent such manipulation, the L-scale was designed to detect and provide information on responses intentionally distorted or skewed toward socially desirable traits (Furnham, 1986). Although L-scale can detect “faking” subjects, limitation remains in accurately discerning every faking subject from honest subjects (Elliot et al., 1996). In addition, since item contents and anchors are pre-determined, test respondents cannot provide detailed information beyond test items (Arntz et al., 2012). According to Paulhus and Vazire (2007), this is especially evident in dichotomous response formats (e.g., Yes-No, True-False, and Agree-Disagree). Finally, test bias due to absolute or random responding also remains a critical issue in test administration (Holden et al., 2012; Al-Mosaiwi and Johnstone, 2018).

Structured or semi-structured clinical interview methods are utilized in personality assessment and diagnosis since it allows more information than the self-reported multiple choice questionnaires, and participants are less likely to hide or distort their responses. These interview methods can also increase the reliability of personality disorder diagnosis in compliance to the diagnostic criteria (Wood et al., 2002). Clinicians can identify discrepancies found in self-reported tests and obtain additional information on responses through follow-up questions, which is essential for diagnosing personality disorders (Samuel et al., 2013). Especially, the Structured Interview for the FFM (SIFFM; Trull and Widiger, 1997) and the Structured Clinical Interview for the DSM-5 Alternative Model for Personality Disorder (SCID-5-AMPD) are representative interview instruments that evaluate personality traits in terms of dimensional aspect. These interviews may better describe behavioral symptoms and diagnostic criteria in a systematic and standardized manner because of their superior assessment of observable behavioral symptoms (Hopwood et al., 2008). However, it is important to note that semi-structured interview requires a lot of time and manpower. Also, evaluation relying on clinician’s judgment may cause diagnosis bias or problems with reliability.

#### Machine Learning and Natural Language Processing in Psychology

With advancements in computer technology, new attempts have been made to analyze psychological traits through computer programming and to predict them quickly, efficiently, and accurately. Especially with the rise of Machine Learning (ML), Deep Learning (DL), and Natural Language Processing (NLP), researchers in the field of psychology are widely adopting NLP to assess psychological construct or to predict human behaviors. ML and DL mainly focus on developing algorithms to discover certain patterns and predict new data accumulated from prior experiences, learned by computer programs through previously performed similar tasks. ML and DL enable researchers to identify independent variables, which were previously under-recognized, and to handle tremendous data. Natural language processing (NLP), a branch of ML research and applications, incorporates computer programming that automatically understand and analyze natural language text. NLP researchers have developed appropriate tools and techniques to enable computer systems to understand and manipulate natural language to perform desired tasks (Chowdhury, 2003). NLP also makes it possible to quantify and analyze qualitative text data.

According to previous studies, some researchers applied ML and NLP to measure and predict psychological traits such as personality and psychiatric disorders. For example, Al Hanai et al. (2018) attempted to predict depression by developing an automated depression-detection algorithm that learns from a sequence of questions and answers. Jayaratne and Jayatilleke (2020) sought to predict one’s personality as an indicator of job performance and satisfaction using the textual content of interview answers. Also, recent studies aim to identify psychotic symptoms and improve the efficient detection of individuals at risk for psychosis by applying NLP to language data (Chandran et al., 2019; Corcoran and Cecchi, 2020; Irving et al., 2021). When Park et al. (2015) utilized ML and NLP to build the open-vocabulary language model with Facebook posts, the model appropriately predicted the participants’ personality based on the FFM.

Likewise, studies attempting to predict and diagnose individual psychological characteristics using ML and NLP techniques are gradually increasing in the field of psychology and mental health. This not only increases efficiency, but also reduces the influence of human bias of the existing measurements (Oswald et al., 2020). However, it is notable that the prior studies still have limitations and many areas need to be supplemented. First, there is a lack of connections between the work being performed in computer science and that of psychology (Stachl et al., 2020). Previous research works in computer science fall short in providing in-depth personality assessment and interpretation, only citing the psychological literature with respect to dependent (target) variables like personality inventories. The explanatory power of the results is questionable in that it overlooked the importance of personality theories. For instance, some studies not only applied non representative SNS profiles (Amichai-Hamburger and Vinitzky, 2010; Lima and De Castro, 2014) or consumption data (Gladstone et al., 2019) as independent (predictor) variables for personality prediction, but results of the MBTI (Myers-Briggs Type Indicators) test, which classify human characteristics into four domains, were also used as target variables despite lacking sufficient theoretical basis (Cui and Qi, 2017; Gjurković and Šnajder, 2018; Sönmezöz et al., 2020). Using these variables makes it possible for convenient and quick big data collection and provides simple labeling for ML, but it does not give an adequate explanation for the predicted results. Second, studies published in the field of clinical and personality psychology often show potential overfitting problems caused by smaller datasets (Yarkoni and Westfall, 2017) and raise concerns about questionable modeling practices (Mønsted et al., 2018). Lastly, it should also be noted that compared to Western countries, studies on language-personality interconnectedness in the Eastern countries and cultures have been relatively less reported.

### Purpose

Traditional approaches using self-report multiple choice questionnaires and recent approaches using machine learning both have their strengths and limitations in personality assessment. Although ML allows faster mappings between data, the results are meaningful only when explanations for complex multidimensional human personality can be provided based on theory. The current study aims to examine the relationship between the FFM personality constructs, psychological distress, and natural language data, overcoming the lack of connection between the field of computer science and psychology. We developed the interview (semi-structured) and open-ended questions for the FFM-based personality assessments, specifically designed with experts in the field of clinical and personality psychology (phase 1). Developed interview questions that could extract linguistic data reflecting personality were formulated and will further be analyzed by NLP. This will help us acquire essential text data to increase the efficiency of ML analysis at the final research stage. We will collect the personality-related text data using the interview questions and extract linguistic features predicting the FFM of personality and psychological distress such as depression, anxiety, and risks for suicidality (phase 2). Finally, we will develop algorithm models that can predict personality with text data. We expect that the newly developed models will better predict personality compared to the traditional assessments. This will bring about important implications in that the research approach is not only exploratory but also theory driven, with sufficient amount of text data provided.

### Hypotheses

We hypothesize that the extracted linguistic feature (1) would identify individuals’ personality traits based on the FFM, having significant correlations with the self-reported FFM personality inventories and (2) would discern a linguistic marker of psychological distress. Also, (3) qualitative differences between the text data obtained from the video interview and the text data obtained from the online survey will be examined through an exploratory method.

## Method and Analysis

### Study Design and Procedure

**Figure 1:**
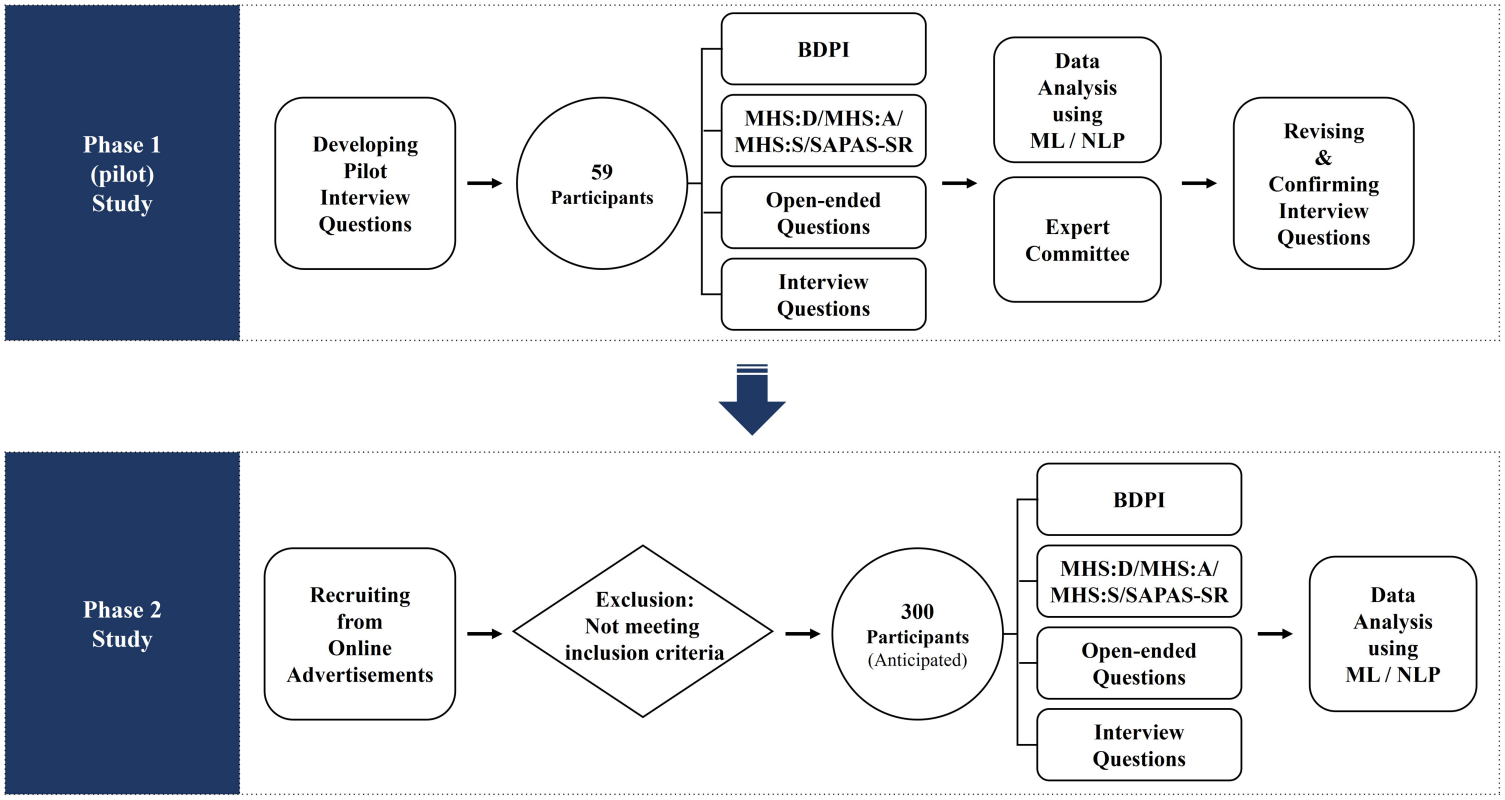
Study design flow.

The current research consists of two phases to provide more explanatory power. In phase I, we conducted a pilot study to develop the semi-structured interview questions for the FFM of personality. In Phase II, the interview for the FFM of personality developed in phase I will be applied in conducting data collection to predict personality and psychological distress (study design and procedure are shown in Figure 1). All the courses of this study will be approved by Korea University’s Institutional Review Boards (IRB). Data will be collected by an online platform considering the COVID-19 pandemic. Finally, the current study will not include any intervention such as pharmacotherapy or psychotherapy.

#### Phase 1 (Pilot) Study

A pilot study was conducted as a process for developing “semi-structured interview” questions based on the FFM of personality. The study was carried out for a year starting from September 2020. First, a preliminary question pool of 66 items was generated by licensed clinical psychologists, social and personality psychologists, psychometricians, and experts in human resource departments. All questions were designed to measure 10 domains and 33 sub-facets based on Costa and McCrae’s Five-Factor model (McCrae and Costa, 1996). Then, we conducted a primary data collection on 59 participants using the 29 items selected through several additional review processes made by experts. All responses collected were once again empirically evaluated for adequacy from an external expert committee, consisting of psychologists with expertise in personality and psychopathology. In particular, differential validity, ambiguity in expressions, and the intention of the question were carefully considered. Furthermore, additional analysis based on ML and NLP for the text data was administered. At this stage, we applied information theory to identify the entropy of words that are highly correlated with specific personality traits, and Latent Dirichlet Allocation (LDA) was used to exploratorily analyze what kind of words related to which certain subjects are frequently used by people. Through these experts’ feedback and computing analysis process, the existing 29 questions were revised and finalized as a total of 18 questions.

#### Phase 2 Study

In phase 2 study, we will collect data using a semi-structured interview, developed in a pilot study and self-report inventories. Phase II started in November, 2021 and will be completed in August, 2023. Participants will first be asked to respond to the self-report questionnaire about depression, anxiety, suicide risk, and personality *via* online survey platform (Qualtrics). Then, they will participate in a semi-structured interview session with the researcher in an online meeting or chatting platform. All responses of the participants to the interview questions were stored and analyzed in the form of text. To extract text features, text data from the interview and the online survey will be preprocessed using morphological analysis and analyzed by applying the NLP and ML model. The results of the online survey including demographic data, depression, anxiety, suicidality, and personality inventories will be investigated together in the model to predict individuals’ personality based on the FFM and the level of psychological distress. The moderating role of qualitative differences of linguistic information, in terms of written text and transcribed speech, in the effects of personality on language patterns or expression needs to be further investigated. To explore this, the same questions will be asked to participants in both online survey (text submission) and online video interview (speech transcribed into text).

#### Participants

Participants will be recruited from online or local advertisements posted in university communities or job search websites. All participants will be provided with written informed consent before participating in the study. The inclusion criteria are (1) being over 18 years and (2) fluent in Korean language. The participants (1) who have a history of brain surgery or (2) intellectual disability will be excluded. A total of 59 participants were recruited in Phase 1, and in Phase 2, we will collect data from 300 (anticipated) Korean adults using a convenient sampling method. This number is considered appropriate in that several similar studies (Hirsh and Peterson, 2009; Arntz et al., 2012; Al Hanai et al., 2018), which directly collected data (not based on big data) from about 100 to 400 people, reported appropriate results.

### Measures

#### Online Survey

Participants will complete a battery of questionnaires designed to assess depression, anxiety, suicidality, personality disorders, personality characteristics, and data on demographic information. In addition, open-ended questions about individuals’ personality will be asked and collected.

#### Bright and Dark Personality Inventory

Bright and Dark Personality Inventory (BDPI; Kim et al., 2020) will be used to assess personality traits. The BDPI is a 173-item multidimensional personality inventory, which is a self-report measure with a 4-point Likert scale (1 = strongly disagree; 4 = strongly agree). BDPI was developed based on Five Factor Model of personality (McCrae and Costa, 1996). BDPI measures personality in five-factor traits and maladaptive traits. Since BDPI measures both adaptive and maladaptive personality traits dimensionally, one’s personality can be described in more detail than traditional approaches which only measured adaptive or maladaptive traits of personality. This approach considering bipolarity of personality can be used to measure one’s personality in various settings from normal to clinical settings (Lee et al., 2019; Kim et al., 2020). The five-factor adaptive personality traits include extraversion, agreeableness, conscientiousness, openness, and emotional stability, and the maladaptive personality traits include detachment, egocentrism, disinhibition, psychoticism, and negative affectivity. BDPI has total of 10 personality traits and each trait consists of three to four sub-facets. Each personality trait and facet will have its own score, which will be converted into T-score.

BDPI was psychometrically validated including Item Response Theory, reporting adequate reliability and validity (Lee et al., 2019; Kim et al., 2020). Kim et al. (2020) reported that Cronbach’s alpha coefficient for adaptive personality scales was 0.924, ranging from 0.714 (conscientiousness) to 0.922 (extraversion) and maladaptive personality scales was 0.960, ranging from 0.848 (psychoticism) to 0.896 (egocentrism). Intraclass correlation coefficient was 0.731 and 0.707, respectively, for adaptive and maladaptive personality scales (Kim et al., 2020).

#### Mental Health Screening Tool for Depressive Disorders

Mental Health Screening Tool for Depressive disorders (MHS:D; Yoon et al., 2018) will be used to assess depressive symptoms. The MHS:D is a 12-item self-report measure, with a 5-point Likert scale (0 = never, 4 = most of the time). Mental Health Screening Tools for depression, anxiety, and suicide risk were developed and validated for Korean adults. MHS:D was developed and validated using structured clinical interview and IRT (Yoon et al., 2018). Weights calculated using IRT will be reflected on total score. In the previous study, Yoon et al. (2018) reported that Cronbach’s alpha coefficient was 0.99. Total score of MHS:D showed positive correlations of 0.74, 0.78, and 0.70 with those of CES-D, PHQ-9, and BDI-II. MHS:D showed 0.92 of sensitivity and 0.94 of specificity for depressive disorder diagnosed with M.I.N.I (Lecrubier et al., 1997) at cut-off point of 13.

#### Mental Health Screening Tool for Anxiety Disorders

Mental Health Screening Tool for Anxiety Disorders (MHS:A; Kim et al., 2018) will be used to assess anxiety symptoms. The MHS:A is a 11-item self-report measure, with a 5-point Likert scale (0 = never, 4 = most of the time). MHS:A was developed and validated using IRT (Kim et al., 2018). Weights calculated using IRT will be reflected on total score. In the previous study, Cronbach’s alpha coefficient was 0.92. Total score of MHS:A showed positive correlations of 0.821, 0.653, and 0.821 of total scores of GAD-7, PSWQ, and BAI, respectively (Kim et al., 2018). MHS:A showed 0.795 of sensitivity and 0.937 of specificity for anxiety disorders at cut-off point of 25, when it showed 0.869 of sensitivity and 0.972 of specificity for GAD at cut-off point of 27.

#### Mental Health Screening Tool for Suicide Risk

Mental Health Screening Tool for Suicide Risk (MHS:S; Yoon et al., 2020) will be used to assess suicide risk. The MHS:S is a 4-item self-report measure, with a 5-point Likert scale (0 = never, 4 = always). MHS:S was developed and validated using IRT (Yoon et al., 2020). Weights calculated using IRT will be reflected on total score. In the previous study, Cronbach’s alpha coefficient was 0.82 for both paper-based and online-based MHS:S. The optimal cut-off scores for risk positive and high risk group were total scores of 1 and 3, respectively (Yoon et al., 2020).

#### Self-Report Standardized Assessment of Personality-Abbreviated Scale

Self-report Standardized Assessment of Personality-Abbreviated Scale (SAPAS-SR) is a self-report version of SAPAS, which is an interview for screening personality disorder (Moran et al., 2003; Choi et al., 2015). It is an 8-item self-report measure, with a dichotomous scale (“Yes” or “No”). The scores on each scale are summed to obtain a total score. Cut-off score of Korean version of SAPAS-SR is 4 of 8, with 67.2% of patients with personality disorders were correctly classified with cut-off score of 4. In previous study, Cronbach’s alpha coefficient for SAPAS scales was 0.79 (Choi et al., 2015).

#### Open-Ended Questions: Self-Description and Adjectives

Participants will be asked to describe their own personality in 300 characters in Korean. They will be also requested to write down six adjectives that describe themselves and another six adjectives which are important to them.

#### Interview Questions Developed for Personality Assessment

Interview questions will be administered to collect linguistic data for personality assessment. Interview questions designed to measure personality assessment consist of 18 questions about the adaptive personality traits (extraversion-introversion, agreeableness, conscientiousness, openness, and emotional stability) and the maladaptive personality traits (detachment, egocentrism, disinhibition, psychoticism, and negative affectivity) and 33 sub-facets based on the FFM (e.g., [Extraversion] “how do you want to spend your time for your routine daily hours?”). Final questionnaires were developed, revised, and confirmed in the pilot study (phase 1). Participants will be instructed to answer the questions reflecting their thoughts and ideas. There will be no time limit for the participants to answer. To exclude the impact of the interviewer on the responses, additional questions will be presented in a pre-determined manner.

### Data Analysis

**Figure 2:**
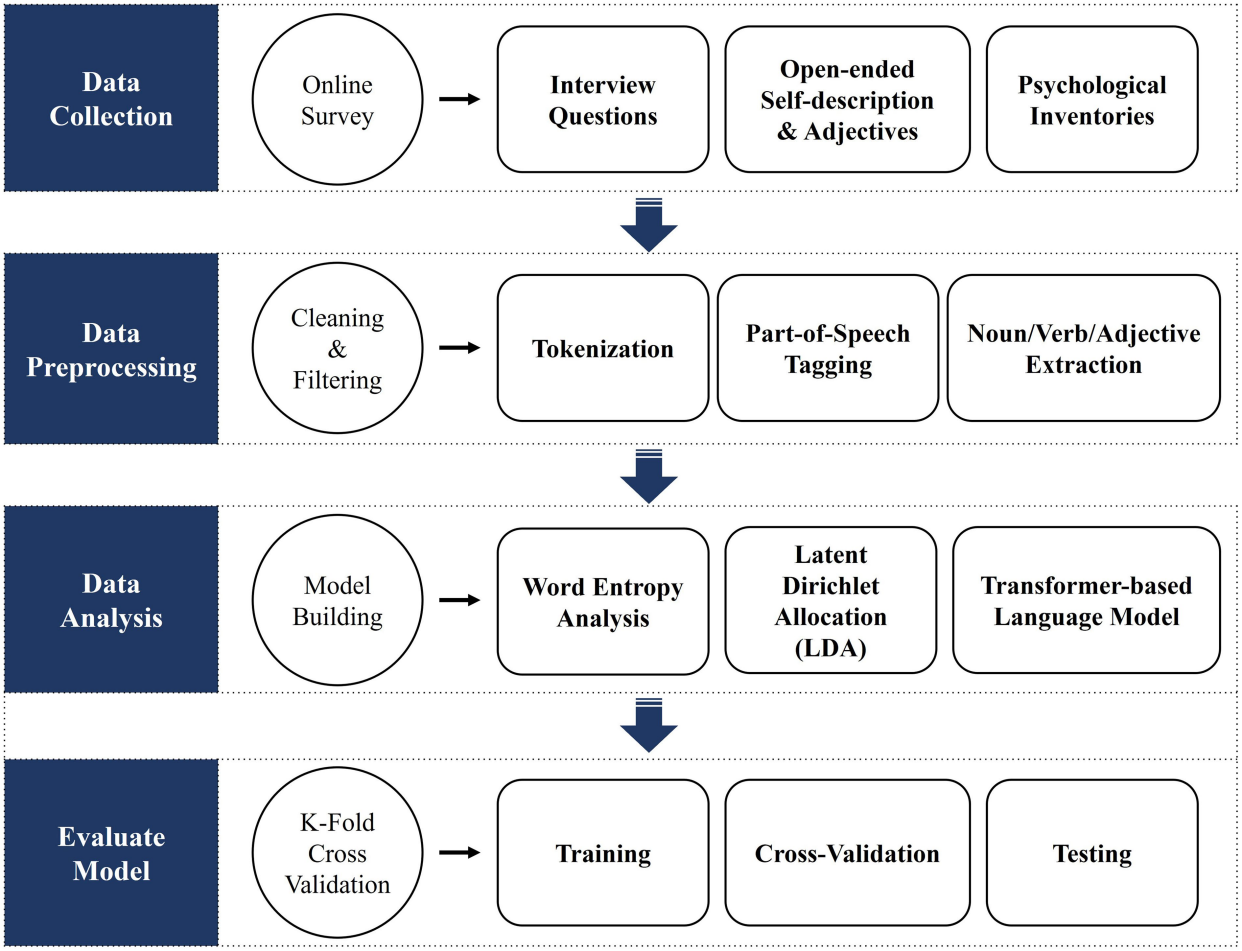
Data analysis plan.

In this study, we will analyze textual data and explore its associations with personality traits through the following analysis plans: (1) Data preprocessing, (2) LDA (Latent Dirichlet Allocation), (3) Transformer-based Korean Language Model, and (4) Training, Cross-validation, and Testing. Python language and Gensim library are planning to be used in the analysis (see Figure 2).

#### Data Preprocessing

After collecting the linguistic data for personality assessment, the data will be cleaned and filtered on the sentence units for analysis. In this step, we will use regular expressions (tokenization) to exclude other punctuation marks and symbols, and then perform part-of-speech tagging will be performed to extract only nouns, verbs, and adjectives which are used as input variables for the prediction model.

#### Latent Dirichlet Allocation

Latent Dirichlet Allocation is an unsupervised statistical language model which enables the discovery of latent topics in unlabeled data (Andrzejewski and Zhu, 2009). By extracting the additional characteristics from the documents, it can be used to supplement the inputs to machine learning and clustering algorithms (Campbell et al., 2015). This algorithm infers variables based on the words from the text data and generates topics for analyzing associations with personality traits. In other words, we will search for topics that can aggregate a large number of words contained in the data collected through LDA and select meaningful topics among them.

#### Transformer-Based Korean Language Model

Transformer-based pretrained language models have enabled neural network models to leverage raw textual data. After training the model with a large amount of unlabeled data in advance, transfer learning using the labeled data can be performed (Devlin et al., 2018). The current study will utilize a transformer-based language model, additionally trained on Korean text. This will go through the process of learning, using text data obtained through the interview (unlabeled) and personality profile obtained through BDPI (labeled). Specifically, the embedding vectors of the sentences collected in the interview are extracted from the pretrained language model, and the scores of the measured personality characteristics are examined to explore words, sentences, and linguistic characteristics highly related to specific personality characteristics. As a result, the transformer model allows the discovery of sentence characteristics that can distinguish personality.

#### Training, Cross-Validation, and Testing

A 75% of the total data will be used for training and cross-validation, and the remaining 25% will be used to evaluate the performance of the trained model (the specific ratio may change depending on the final data size). The training dataset learns the process of finding answers through features, and the cross-validation dataset goes through assessing and comparing learning algorithms. Through the Testing process, we will identify the best fitting classifier and the best model.

## Anticipated Results

### Primary Outcome

Our primary objective is to identify specific linguistic features that correlate with individuals’ personality traits. In particular, we expect that the level of each factor that the FFM describes discovers and classifies linguistic variables that are highly relevant to high or low populations. In addition, we will extract text features that are helpful for predicting personality and apply them in machine learning algorithms to develop a Machine Learning Classification Model of the personality traits based on the FFM. We will examine predictive validity using data obtained from the interview questions as independent variables and individuals BDPI scores as dependent variables. This will add some evidence for the precision of algorithms using natural language processing to predict the ones from the traditional self-report personality questionnaire.

### Secondary Outcome

We aim to detect linguistic markers of psychological distress including depressed symptoms and anxiety symptoms. In particular, words or language characteristics that highly reveal psychological distress in interview contents related to maladaptive facets or negative affectivity. This will enable distress to be quickly and accurately detected and diagnosed through an interview.

### Exploratory Outcomes

Lastly, we hypothesized that there are qualitative differences between the text data obtained from the video interview and the text data obtained from the online survey. Responses to the same question obtained through video interview and online survey were compared and analyzed to see differences in the quality of information provided by face-to-face or non-contact method.

## Discussion

To the best of the author’s knowledge, this will be the first study to predict the FFM-based personality through machine learning technology, using both top-down method, based on personality theory and bottom-up approach, based on the data. Validity will be greater than previous studies in that interview questions are directly established on the FFM theory and that responses are analyzed through ML and NLP. Unlike this study, several studies in the past have used data lacking representativeness, such as Twitter (Quercia et al., 2011) or Facebook (Youyou et al., 2015), to evaluate personality. Correlation and predictive power can be reached by chance. However, it is very insufficient and error-prone to explain complex psychological characteristics such as personality without notable evidence. In other words, since such data are very limited, unexpected inferences can often be made from seemingly random data. But in the field of psychology, presenting a basis for the inference is essential. In this regard, this study will be able to provide evidence and explanation through the FFM.

In addition, the personality evaluation model and algorithm to be developed through this study may reveal better performance than the existing self-report multiple choice questionnaires or clinical interview measurements such as SCID-5-PD or SIFFM. The reason is that compared to the existing multiple choice type tests, questions are rich in information and are difficult to intentionally fake or deceive. Also, accurately predicting personality through statistical modeling and algorithms can reduce the inefficiency of one-on-one interviews while providing solutions to bias or reliability issues caused by relying on the clinician’s personal judgment. Moreover, this study can improve heterogeneity, comorbidity, misdiagnosis, stigma, or labelling problems since personality will be evaluated and diagnosed based on a dimensional approach of FFM, instead of the categorical approach used in the existing clinical field.

On the contrary, many existing studies were conducted on participants who spoke English as their mother tongue (Wongkoblap et al., 2017; Shatte et al., 2019; Le Glaz et al., 2021), and Korean-based studies using the appropriate analysis methods were very limited. Specifically, Calvo et al. (2017) mentioned that studies applying NPL in mental health mostly consist of English-speaking participants, because predicting psychological characteristics by applying NLP to non-English languages is an unexplored area. This study can serve as a starting point for future studies that attempt to predict psychological characteristics by analyzing and learning Korean rather than English. However, problems arise in that psychological data are very sensitive and that it is difficult to obtain large amounts of information rapidly. Due to security issues, a lot of time and effort is needed in collecting large amounts of data unlike the other fields where pre-labeled information can be easily obtained through open source. Nevertheless, if data are collected and actively shared along with strict security management, sophisticated models and algorithms can be refined and the use of computer technology in the field of psychology can be further developed.

## Conclusion

Unlike other fields that simply analyze large amounts of data, human psychology, mental characteristics, and personality characteristics require more explanations. We are confident that this will be a representative study meeting the criteria. We believe that this study will be of great interest to future studies seeking to improve the problems of the psychological evaluation methods through the advantages of using computers compared to humans, as well as combining advanced technologies such as psychology, machine learning, big data, and AI.

## Data Availability Statement

The raw data supporting the conclusions of this article will be made available by the authors, without undue reservation.

## Ethics Statement

The studies involving human participants were reviewed and approved by the local Institutional Review Board (IRB) of Korea University. The patients/participants provided their written informed consent to participate in this study. The same ethical protocols will apply to ongoing research related to this study.

## Author Contributions

JJ, SY, JC, and K-HC contributed to the conception and design of the study. K-HC administrated the overall study process. JC designed the overall analysis procedure and contributed to the revision of the semi-structured interview *via* pilot data analysis. JJ and SY contributed to acquisition of the phase 1 (pilot) data. JJ, SY, GS, and K-HC designed and revised the semi-structured interview. JJ, SY, and GS wrote the first draft of the manuscript. JJ, MK, JC, and K-HC contributed to editing the draft of the manuscript. All authors contributed to the article and approved the submitted version.

## Funding

This work was supported by the National Research Foundation of Korea (NRF) grant funded by the Korea government (MSIT; no. NRF-2020R1A2C2099665).

## Conflict of Interest

The authors declare that the research was conducted in the absence of any commercial or financial relationships that could be construed as a potential conflict of interest.

## Publisher’s Note

All claims expressed in this article are solely those of the authors and do not necessarily represent those of their affiliated organizations, or those of the publisher, the editors and the reviewers. Any product that may be evaluated in this article, or claim that may be made by its manufacturer, is not guaranteed or endorsed by the publisher.
